# The Effects of Chlorhexidine, Essential Oil‐Based, and Stannous Fluoride Mouthrinses on the Color Stability and Surface Roughness of CAD/CAM Ceramics

**DOI:** 10.1002/cre2.70469

**Published:** 2026-10-06

**Authors:** Fateme Ghasemian Gorji, Fariba Ezoji, Ghazaleh Ahmadizenouz, Seyedali Seyedmajidi

**Affiliations:** ^1^ Student Research Committee Babol University of Medical Sciences Babol I.R. Iran; ^2^ Dental Materials Research Center, Health Research Institute Babol University of Medical Sciences Babol I.R. Iran; ^3^ Oral Health Research Center, Health Research Institute Babol University of Medical Sciences Babol I.R. Iran

**Keywords:** CAD/CAM ceramics, color stability, mouthrinse, surface roughness

## Abstract

**Objectives:**

The long‐term esthetic performance of CAD/CAM ceramic restorations may be influenced by repeated exposure to therapeutic mouthrinses. This study evaluated the effects of chlorhexidine, essential oil‐based, and stannous fluoride mouthrinses on the color stability (Δ*E*) and surface roughness (*R*_a_) of glazed and polished CAD/CAM ceramics.

**Materials and Methods:**

In this in vitro experimental study, 320 specimens fabricated from four CAD/CAM ceramic materials (Vita Mark II, IPS e.max CAD, CAD, Celtra Duo, and high‐translucency zirconia) were randomly assigned to glazed or polished surface treatment groups and immersed daily for 20 min in chlorhexidine, essential oil‐based, stannous fluoride mouthrinses, or distilled water (control) for 30 days. Color stability was assessed using spectrophotometry based on the CIE Lab* color system, and surface roughness was measured using laser profilometry. Data were analyzed using one‐way ANOVA followed by Tukey's post hoc test (*α* = 0.05).

**Results:**

The effects of therapeutic mouthrinses on color stability and surface roughness varied according to ceramic material and surface finish. The greatest color change was observed in polished IPS e.max CAD immersed in the essential oil‐based mouthrinse (Δ*E* = 3.69), whereas the lowest Δ*E* was recorded for glazed Celtra Duo immersed in stannous fluoride (Δ*E* = 1.59). Polished specimens consistently exhibited greater increases in surface roughness than glazed specimens across all ceramic materials. Significant differences in Δ*E* and/or *R*_a_ were identified according to ceramic type, mouthrinse composition, and surface finish (*p* < 0.05).

**Conclusion:**

The effects of therapeutic mouthrinses on CAD/CAM ceramics depend on both ceramic composition and surface finish. Essential oil‐based mouthrinse produced the greatest color changes in several ceramic materials, whereas glazed surfaces generally demonstrated greater resistance to surface roughness changes than polished surfaces. These findings highlight the importance of considering both material selection and surface finishing when recommending therapeutic mouthrinse use for patients with CAD/CAM ceramic restorations.

## 1 Introduction

Advances in CAD/CAM ceramic materials have substantially expanded the clinical applications of esthetic dentistry owing to their favorable optical properties, biocompatibility, and mechanical performance (Zarone et al. 2019). Nevertheless, their long‐term performance can be compromised by daily contact with oral hygiene agents, including therapeutic mouthrinses, which may alter optical and surface characteristics (Hazar and Hazar 2024).

Color stability is vital for clinical efficacy, as noticeable discoloration (Δ*E* > 3.3) often results in patient dissatisfaction (Bozkaya Bilgin and Tekçe 2025). Therapeutic mouthrinses are widely used as adjuncts to mechanical plaque control and differ considerably in their chemical composition, mechanisms of action, and clinical indications. Chlorhexidine is widely regarded as the gold‐standard antimicrobial mouthrinse because of its broad‐spectrum antibacterial activity and prolonged substantivity; however, its long‐term use has been associated with extrinsic staining of both teeth and restorative materials (Van Strydonck et al. 2012). Essential oil‐based mouthrinses exert antimicrobial activity through phenolic compounds and frequently contain alcohol, which may adversely affect the surface integrity of glass‐containing restorative materials following prolonged exposure. Stannous fluoride mouthrinses are widely recommended for caries prevention and erosion control; however, the presence of tin ions and the acidic pH of some formulations may contribute to surface alterations and discoloration of restorative materials. These compositional differences may influence the interaction between mouthrinses and restorative materials, potentially affecting their optical and surface properties (Radzki et al. 2022; Melo et al. 2025). The Commission Internationale de l'Éclairage (CIE) L*a*b* color system is a three‐dimensional color space in which L* represents lightness, a* the green–red axis, and b* the blue–yellow axis. Overall color difference (Δ*E*) is calculated from these coordinates and is widely used to evaluate clinically relevant color changes in restorative materials (Gawriołek et al. 2012). Mouthrinses containing pigments, alcohol, or metal ions (e.g., stannous fluoride) can exacerbate discoloration via oxidative processes or surface erosion (Radzki et al. 2022; Melo et al. 2025; Kader et al. 2025).

In addition to color stability, surface roughness (*R*_a_) is another important determinant of the clinical performance of ceramic restorations, as increased roughness promotes plaque accumulation, bacterial adhesion, and wear. Profilometric analyses indicate that chemical exposure modifies *R*_a_, yet evidence on mouthrinse effects remains inconsistent (Younes et al. 2024). For example, alcohol‐containing essential oil‐based mouthrinses may promote hydrolytic degradation of the glass matrix, whereas chlorhexidine may interact with silica‐rich ceramic surfaces through its cationic components, potentially altering surface characteristics (Radzki et al. 2022; Lee et al. 2020; James et al. 2017).

Common CAD/CAM ceramics differ considerably in their composition, microstructure, and clinical performance. IPS e.max CAD (lithium disilicate) is widely used because of its excellent esthetics, translucency, and favorable mechanical properties, although its glass‐rich matrix may make it more susceptible to chemical degradation under prolonged exposure. Celtra Duo (zirconia‐reinforced lithium silicate) combines improved strength with favorable optical properties through zirconia reinforcement. However, its long‐term chemical stability following exposure to fluoride‐containing agents remains insufficiently investigated. Vita Mark II, a feldspathic ceramic, is valued for its enamel‐like esthetics and excellent machinability but exhibits lower fracture resistance than glass‐ceramic and zirconia‐based materials. High‐translucency zirconia provides superior mechanical strength and fracture resistance while offering improved esthetics compared with conventional zirconia, although its translucency remains lower than that of glass ceramics (Zarone et al. 2019; Daryakenari et al. 2019; Krejci et al. 1999).

Although several studies have investigated beverage‐induced staining of ceramic materials (Dos Santos et al. 2017; Palla et al. 2018), systematic comparative evaluations of therapeutic mouthrinses on CAD/CAM ceramics remain limited. Most existing research focuses on short‐term exposure, individual materials (e.g., zirconia or lithium disilicate alone), or specific mouthrinse types, with inconsistent findings regarding surface treatments (glazed vs. polished) and prolonged protocols. Recent studies have reported changes in hardness, surface roughness, and color following exposure to antiseptic or fluoride‐containing mouthrinses. However, direct comparative evaluations of chlorhexidine, essential oil‐based, and stannous fluoride mouthrinses across multiple CAD/CAM ceramic materials remain scarce, particularly under prolonged immersion protocols intended to simulate long‐term clinical use (Alpkilic et al. 2021; Esfahanizadeh et al. 2024; Hazar and Hazar 2024; Melo et al. 2025; Kader et al. 2025).

Therefore, this in vitro study aimed to evaluate the effects of chlorhexidine, essential oil‐based, and stannous fluoride mouthrinses on the color stability (Δ*E*) and surface roughness (*R*_a_) of four CAD/CAM ceramic materials with glazed and polished surface finishes under standardized experimental conditions. The findings may help identify clinically relevant esthetic risks, support evidence‐based recommendations for restorative material selection and maintenance, and provide a foundation for future clinical investigations.

## 2 Materials and Methods

### 2.1 Study Design

This in vitro experimental study adopted a factorial design (4 ceramic types × 2 surface treatments × 4 immersion groups; *n* = 10 per subgroup, total 320 specimens) to evaluate the effects of mouthrinse type, ceramic material, and surface finish on color stability (Δ*E*) and surface roughness (*R*_a_) of CAD/CAM ceramics. The sample size (*n* = 10 per subgroup) was selected based on previously published in vitro studies investigating the effects of mouthrinses on the color stability and surface properties of CAD/CAM ceramic materials using comparable experimental designs (Soygun et al. 2017; Alpkilic et al. 2021). The study followed standardized protocols for specimen preparation, mouthrinse immersion, color assessment, and surface roughness measurements based on previously published in vitro methodologies to ensure methodological consistency and reproducibility (Festuccia et al. 2012; Derafshi et al. 2017; Zakir et al. 2020; Alkhudair et al. 2022).

### 2.2 Sample Preparation

Four commercially available CAD/CAM ceramic materials were selected:

1. Vita Mark II (feldspathic ceramic; VITA Zahnfabrik, Germany),
2. IPS e.max CAD (lithium disilicate; Ivoclar Vivadent, Germany),
3. Celtra Duo (zirconia‐reinforced lithium silicate; Dentsply Sirona, Germany),
4. Translucent zirconia (high‐translucency zirconia; Dental Direkt, Germany).

Raw blocks were sectioned into 10 × 10 × 2 mm specimens using a diamond cutting disc (0.3 mm thickness; NemoFnavaran Pars, Mashhad, Iran) under continuous water cooling. A total of 80 specimens were prepared per ceramic type, ensuring uniform dimensions and specifications. To mimic post‐machining surface conditions in CAD/CAM systems, specimens were sequentially polished with silicon carbide abrasive papers (600‐, 800‐, and 1000‐grit) under running water.

### 2.3 Group Allocation and Surface Treatments

Specimens were randomly assigned to two equal groups (*n* = 40 per group): glazed or polished.

### 2.4 Glazing Procedure

Glazing was performed in a VITA ceramic furnace (Certified VITA Furnaces, Bad Säckingen, Germany) following previously published glazing protocols for CAD/CAM ceramic materials to standardize the surface treatment procedure (Martínez‐Gomis et al. 2003; Alp et al. 2018; Singh et al. 2017).

- For Vita Mark II: Glaze material (VITA Glaze LT, VITA Zahnfabrik) was applied, followed by a four‐stage firing: preheating at 600°C for 4 min, heating at 75°C/min to 900°C, and holding at 900°C for 1 min (total firing time: 9 min).
- For Celtra Duo: Universal Spray Glaze (Dentsply Sirona) was applied, with firing in a vacuum furnace: preheating at 480°C for 4 min, heating at 60°C/min to 820°C, and holding for 1 min (total: 10 min 40 s).
- For IPS e.max CAD: IPS e.max CAD Crystall Glaze (Ivoclar Vivadent) was applied, with a single‐stage hold at 550°C for 6 min.
- For high‐translucency zirconia: Initial sintering followed the manufacturer's protocol (heating to 900°C for 30 min, then to 1450°C for 120 min, slow cooling to 200°C). Glazing used UniGlaze (Dental Direkt): preheating at 440°C for 2 min, heating at 45°C/min to 815°C, holding for 1 min, and cooling for 3 min.

Final surface adjustments were made with a fine‐grit fissure bur (D+Z Diamant, Germany) to simulate clinical contouring.

### 2.5 Polishing Procedure

For polishing the ceramic samples, a DIAPOL polishing kit (EVE Ernst Vetter GmbH, Germany) was employed. The polishing procedure followed these steps:

1. A blue (smoothing finisher) disk was used initially to create a smooth surface.
2. A pink (pre‐polisher) disk was then used for the initial polishing.
3. Finally, a gray (high‐shine polisher) disk was applied to achieve a high‐gloss finish.

The polishing was performed using a low‐speed handpiece, with each sample polished for 1 min.

For polishing the zirconia samples, a DIACERA polishing kit (EVE Ernst Vetter GmbH, Germany) was used. The process began with a green (pre‐polisher) disk for initial polishing, followed by an orange (high‐shine polisher) disk to attain a glossy surface.

All polishing procedures were performed by the first author under the supervision of an experienced prosthodontist. Performing all polishing procedures by a single operator under direct supervision helped minimize operator‐related variability throughout the study.

### 2.6 Color Evaluation

To standardize color changes, all ceramic samples were selected in the A2 color shade. Color measurements were performed using a VITA Easyshade Advance spectrophotometer (VITA Zahnfabrik, Germany) based on the Commission Internationale de l'Éclairage (CIE) L*a*b* color system, in which L* represents lightness, a* the green–red axis, and b* the blue–yellow axis. Each sample was directly measured with the spectrophotometer, with three readings taken per sample. The average values of L*, a*, and b* from the three measurements were recorded (Festuccia et al. 2012). Before each measurement session, the spectrophotometer was calibrated according to the manufacturer's instructions to ensure measurement accuracy and consistency.

### 2.7 Surface Roughness Evaluation

*R*_a_ (in µm) was measured using a laser profilometer (Nemo Fnavaran Pars, Mashhad, Iran; 1 µm accuracy). A 6 mm^2^ area per specimen was scanned with 100 µm step intervals in X and Y directions. Mean *R*_a_ values were recorded from three tracings (Zakir et al. 2020).

### 2.8 Mouthrinse Exposure

Following baseline evaluations, glazed and polished specimens of each ceramic were randomly allocated to four subgroups (*n* = 10 each). Specimens were stored in artificial saliva (Caphosol, Cytogen Corp., Princeton, NJ, USA) at 37°C and immersed daily for 20 min in 10 mL of one of the following:

1. 0.2% chlorhexidine gluconate (alcohol‐free, pH 5.1; Shahrdarou Co., Tehran, Iran),
2. Essential oil‐based mouthrinse (alcohol‐based, pH 4.2; Listerine Tooth Defense Antic, Johnson & Johnson, Italy),
3. Stannous fluoride mouthrinse (pH 3.8; Meridol, Colgate‐Palmolive, Hamburg, Germany),
4. Distilled water (control group).

Mouthrinses were refreshed daily, and immersion continued for 30 days (simulating ~6 months of 2‐min daily clinical use). Post‐immersion, specimens were rinsed in distilled water for 120 s and air‐dried (Alkhudair et al. 2022; Derafshi et al. 2017). According to commonly accepted clinical thresholds, Δ*E* values below approximately 3.3 are generally considered clinically acceptable, whereas higher values may become visually perceptible and esthetically unacceptable (Bozkaya Bilgin and Tekçe 2025).

### 2.9 Re‐Evaluations

The samples were re‐evaluated for color and surface roughness. The changes in roughness were calculated by subtracting the pre‐immersion values from the post‐immersion values.

Color differences were quantified using the Δ*E* value, which represents the overall color difference between baseline and post‐immersion measurements, calculated from the changes in the L*, a*, and b* coordinates according to the CIE L*a*b* color system, using the following formula:

$$\Delta E=\sqrt{\Delta L^{2}+\Delta a^{2}+\Delta b^{2}}$$

Higher Δ*E* values indicate greater color changes, whereas lower values indicate better color stability. According to commonly accepted clinical thresholds, Δ*E* values below approximately 3.3 are generally considered clinically acceptable, whereas higher values may become visually perceptible and esthetically unacceptable (Bozkaya Bilgin and Tekçe 2025).

### 2.10 Statistical Analysis

Statistical analysis was performed using SPSS version 26. One‐way ANOVA with Tukey's multiple comparison test compared mean changes across mouthrinses within ceramic and surface groups. Independent samples *t*‐tests compared glazed versus polished specimens for each mouthrinse. Significance was set at *p* ≤ 0.05.

## 3 Results

Figure 1 presents the mean color change (Δ*E)* and surface roughness (*R*_a_) values after 30 days of mouthrinse exposure. The distilled water control group exhibited negligible changes (Δ*E* < 0.5 and *R*_a_ < 0.1 µm) and was therefore excluded from the comparative statistical analyses presented in Tables 1, 2, 3, 4.

**Figure 1:**
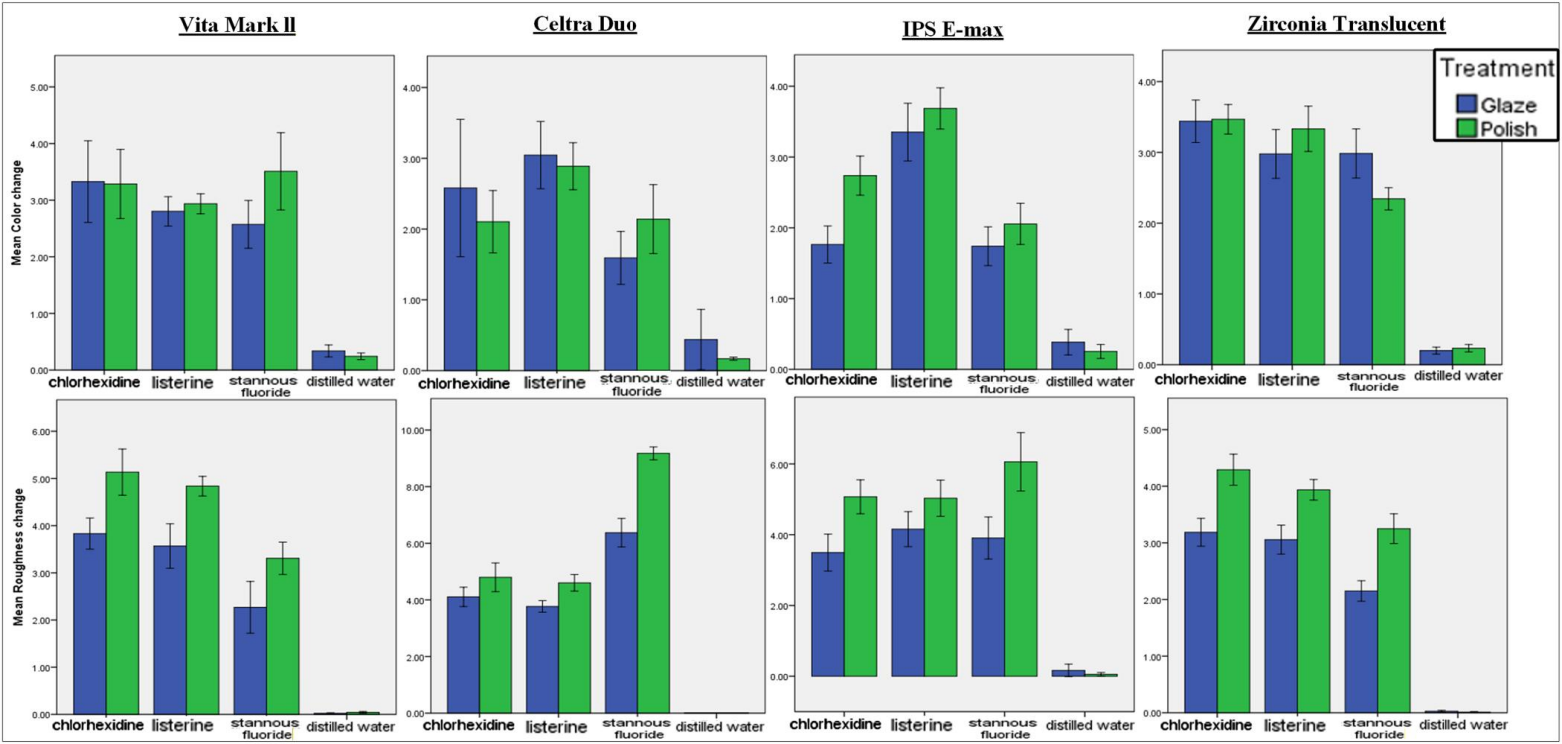
Mean color change (Δ*E*) and surface roughness (*R*_a_) after mouthrinse exposure, stratified by ceramic type and surface treatment (glazed/polished). Error bars represent standard deviation.

**Table 1:** Comparison of mean values for color change and surface roughness of glazed and polished Vita Mark II ceramic samples exposed to different mouthwashes, and between glazed and polished samples within each mouthwash.

Treatment type	Mouthwash				
	Variable change	Chlorhexidine	Listerine	Stannous fluoride	*p* value*
Glazed	Color (Δ*E*)	3.33 (1.01)	2.8 (0.36)	2.57 (0.59)^a^	0.66
	Surface roughness (µm)	3.83 (0.46)^a^ ^B^	3.57 (0.66)^a^ ^B^	2.27 (0.77)^a^ ^A^	**< 0.001**
Polished	Color (Δ*E*)	3.29 (0.85)	2.94 (0.25)	3.51 (0.95)^a^	0.247
	Surface roughness (µm)	5.13 (0.68)^a^ ^B^	4.84 (0.29)^a^ ^B^	3.31 (0.48)^a^ ^A^	**< 0.001**

*Note:* Non‐matching letters indicate a significant statistical difference between the mean of each variable between different mouthwashes. Data are presented as mean (SD).

* ANOVA.

^a^ Indicates a significant statistical difference between the mean of each variable between glazed and polished samples for each mouthwash. Bold *p* values indicate statistically significant differences among the three mouthrinses within the corresponding ceramic and surface‐treatment group (*p* ≤ 0.05).

**Table 2:** Comparison of mean values for color change and surface roughness of glazed and polished Celtra Duo ceramic samples exposed to different mouthwashes, and between glazed and polished samples within each mouthwash.

Treatment type	Mouthwash				
	Variable change	Chlorhexidine	Listerine	Stannous fluoride	*p* value*
Glazed	Color (Δ*E*)	2.58 (1.36)	3.05 (3.66)^B^	1.59 (0.52)^A^	**0.005**
	Surface roughness (µm)	4.11 (0.48)^a^ ^B^	3.77 (0.29)^a^ ^B^	6.37 (0.7)^a^ ^A^	**< 0.001**
Polished	Color (Δ*E*)	2.1 (0.62)^B^	2.89 (0.46)^A^	2.14 (0.68)^B^	**0.01**
	Surface roughness (µm)	4.8 (0.71)^a^ ^B^	4.6 (0.41)^a^ ^B^	9.18 (0.32)^a^ ^A^	**< 0.001**

*Note:* Non‐matching letters indicate a significant statistical difference between the mean of each variable between different mouthwashes. Data are presented as mean (SD).

* ANOVA.

^a^ Indicates a significant statistical difference between the mean of each variable between glazed and polished samples for each mouthwash. Bold *p* values indicate statistically significant differences among the three mouthrinses within the corresponding ceramic and surface‐treatment group (*p* ≤ 0.05).

**Table 3:** Comparison of mean values for color change and surface roughness of glazed and polished IPS e‐max ceramic samples exposed to different mouthwashes, and between glazed and polished samples within each mouthwash.

Treatment type	Mouthwash				
	Variable change	Chlorhexidine	Listerine	Stannous fluoride	*p* value*
Glazed	Color (Δ*E*)	1.76 (0.37)^a^ ^B^	3.35 (0.57)^A^	1.74 (0.38)^B^	**< 0.001**
	Surface roughness (µm)	3.49 (0.73)^a^	4.16 (0.69)^a^	3.91 (0.83)^a^	0.157
Polished	Color (Δ*E*)	2.74 (0.39)^a^ ^A^	3.69 (0.41)^B^	2.06 (0.41)^C^	**< 0.001**
	Surface roughness (µm)	5.07 (0.67)^a^ ^B^	5.03 (0.72)^a^ ^B^	6.06 (1.16)^a^ ^A^	**0.021**

*Note:* Non‐matching letters indicate a significant statistical difference between the mean of each variable between different mouthwashes. Data are presented as mean (SD).

* ANOVA.

^a^ Indicates a significant statistical difference between the mean of each variable between glazed and polished samples for each mouthwash. Bold *p* values indicate statistically significant differences among the three mouthrinses within the corresponding ceramic and surface‐treatment group (*p* ≤ 0.05).

**Table 4:** Comparison of mean values for color change and surface roughness of glazed and polished Zirconia Translucent ceramic samples exposed to different mouthwashes, and between glazed and polished samples within each mouthwash.

Treatment type	Mouthwash				
	Variable change	Chlorhexidine	Listerine	Stannous fluoride	*p* value*
Glazed	Color (Δ*E*)	3.44 (0.42)	2.98 (0.48)	2.98 (0.49)^a^	0.055
	Surface roughness (µm)	3.19 (0.34)^a^ ^B^	3.06 (0.36)^a^ ^B^	2.15 (0.25)^a^ ^A^	**< 0.001**
Polished	Color (Δ*E*)	3.47 (0.29)^B^	3.33 (0.45)^B^	2.34 (0.22)^a^ ^A^	**< 0.001**
	Surface roughness (µm)	4.29 (0.38)^a^ ^B^	3.94 (0.25)^a^ ^B^	3.25 (0.37)^a^ ^A^	**< 0.001**

*Note:* Non‐matching letters indicate a significant statistical difference between the mean of each variable between different mouthwashes. Data are presented as mean (SD).

* ANOVA.

^a^ Indicates a significant statistical difference between the mean of each variable between glazed and polished samples for each mouthwash. Bold *p* values indicate statistically significant differences among the three mouthrinses within the corresponding ceramic and surface‐treatment group (*p* ≤ 0.05).

### 3.1 Vita Mark II Ceramic

Table 1 summarizes the mean Δ*E* and *R*_a_ changes for glazed and polished Vita Mark II specimens across mouthrinses, along with inter‐group comparisons.

No significant differences in color change (Δ*E*) were observed among the tested mouthrinses for either glazed or polished Vita Mark II specimens (*p* > 0.05). In contrast, surface roughness differed significantly among mouthrinses in both surface conditions (*p* < 0.001). Compared with glazed specimens, polished Vita Mark II specimens exhibited significantly greater increases in surface roughness across all mouthrinse groups, whereas a significant inter‐surface difference in Δ*E* was observed only after exposure to stannous fluoride.

### 3.2 Celtra Duo Ceramic

Table 2 presents the corresponding data for Celtra Duo specimens.

Significant differences in both color change (Δ*E*) and surface roughness (*R*_a_) were observed among the tested mouthrinses for glazed and polished Celtra Duo specimens (*p* ≤ 0.01). Compared with glazed specimens, polished specimens showed significantly greater increases in surface roughness across all mouthrinse groups, whereas color change did not differ significantly between surface treatments.

### 3.3 IPS e‐max Ceramic

Table 3 details the findings for IPS e.max CAD.

Significant differences in color change (Δ*E*) were observed among the tested mouthrinses for both glazed and polished IPS e.max CAD specimens (*p* < 0.001). Essential oil‐based mouthrinse produced the greatest color change, whereas surface roughness differed significantly among mouthrinses only in polished specimens (*p* = 0.021). Compared with glazed specimens, polished IPS e.max CAD specimens exhibited significantly greater increases in surface roughness, whereas a significant inter‐surface difference in Δ*E* was observed only after chlorhexidine exposure.

### 3.4 Zirconia Translucent Ceramic

Table 4 summarizes the results for high‐translucency zirconia.

No significant differences in color change (Δ*E*) were observed among the tested mouthrinses for glazed high‐translucency zirconia specimens (*p* = 0.055). In contrast, significant differences in both Δ*E* and surface roughness (*R*_a_) were observed among the tested mouthrinses for polished specimens (*p* < 0.001). Compared with glazed specimens, polished zirconia specimens consistently exhibited greater increases in surface roughness. A significant inter‐surface difference in Δ*E* was observed only in the stannous fluoride group.

## 4 Discussion

This in vitro study evaluated the effects of three commonly prescribed therapeutic mouthrinses—chlorhexidine, an essential oil‐based formulation, and stannous fluoride—on the color stability (Δ*E*) and surface roughness (*R*_a_) of four CAD/CAM ceramic materials with glazed and polished surface finishes. The findings demonstrated that the response of CAD/CAM ceramic materials to mouthrinse exposure was highly dependent on both ceramic composition and surface treatment.

Vita Mark II demonstrated relatively stable color values across all tested mouthrinses despite measurable increases in surface roughness, suggesting that color stability and surface degradation do not necessarily occur concurrently. Similar findings have been reported for feldspathic ceramics, which generally exhibit favorable optical stability despite surface alterations following chemical aging (Soygun et al. 2017; Alpkilic et al. 2021). In contrast, Celtra Duo and high‐translucency zirconia exhibited mouthrinse‐dependent variations in both Δ*E* and *R*_a_, whereas IPS e.max CAD showed greater susceptibility to color change than to surface roughness alterations. These observations are consistent with previous reports indicating that the response of CAD/CAM ceramic materials to chemical exposure is largely governed by differences in ceramic composition, crystalline content, and glass‐matrix characteristics (Melo et al. 2025; Kader et al. 2025).

Although prior research has predominantly investigated beverage‐induced staining on ceramics (Acar et al. 2016; Alp et al. 2018), fewer studies have addressed the impact of therapeutic mouthrinses, despite their frequent and prolonged clinical application (Alpkilic et al. 2021; Dos Santos et al. 2017; Soygun et al. 2017). In the present work, the most pronounced color change occurred in polished IPS e.max CAD exposed to the essential oil‐based mouthrinse (Δ*E* = 3.69), whereas the minimal shift was in glazed Celtra Duo with stannous fluoride (Δ*E* = 1.59). Given that Δ*E* values > 3.3 are generally considered clinically perceptible and unacceptable (Bozkaya Bilgin and Tekçe 2025), certain material‐mouthrinse combinations pose meaningful esthetic risks.

The essential oil‐based mouthrinse consistently produced the greatest color change in both glazed and polished specimens, particularly in IPS e.max CAD. This finding is consistent with previous studies reporting greater discoloration following exposure to alcohol‐containing mouthrinses than chlorhexidine‐based formulations (Jalalian and Ebrahimnejad 2019; Melo et al. 2025). Similarly, Kader et al. (2025) demonstrated that the chemical composition of mouthrinses plays a major role in determining the color stability of CAD/CAM restorative materials. In contrast, Esfahanizadeh et al. (2024) observed no significant differences between chlorhexidine and essential oil‐based mouthrinses, suggesting that variations in ceramic composition, immersion protocols, and mouthrinse formulations may influence the reported outcomes. The greater discoloration associated with essential oil‐based mouthrinses is likely related to their alcohol content and acidic pH, which may promote degradation of the glass matrix and facilitate pigment penetration into surface irregularities. Comparable mechanisms have been proposed in previous in vitro studies evaluating restorative materials exposed to alcohol‐containing mouthrinses (Furtado and Amorim 2019; Lee et al. 2020; Hazar and Hazar 2024).

Material composition appears to play an important role in determining the resistance of CAD/CAM ceramics to mouthrinse‐induced alterations, as their chemical stability is closely related to differences in microstructure and crystalline‐glass composition (Zarone et al. 2019; Melo et al. 2025). The favorable color stability observed for Vita Mark II may be associated with the inherent chemical stability of feldspathic ceramics, whereas the greater susceptibility observed in Celtra Duo and IPS e.max CAD may reflect differences in their glass‐crystalline architecture and their interaction with acidic or alcohol‐containing solutions. Although the stannous fluoride formulation evaluated in this study was acidic, it produced comparatively modest color changes, possibly because tin‐containing formulations can form protective surface deposits that reduce optical alterations (Myers et al. 2019). In contrast, stannous fluoride produced the greatest increase in surface roughness in Celtra Duo in the present study, suggesting that its interaction with zirconia‐reinforced lithium silicate ceramics may differ from that of other mouthrinses and deserves further investigation.

Surface finishing exerted a substantial influence on the optical and surface behavior of all tested ceramic materials. In the present study, polished specimens generally exhibited greater color change than glazed specimens. This finding agrees with previous studies demonstrating that glazed ceramic surfaces are more resistant to staining than polished surfaces because glazing produces a smoother and less porous surface (Alp et al. 2018; Kanat‐Ertürk 2020; Yılmaz et al. 2008). The greater susceptibility of polished specimens may be attributed to residual surface irregularities created during finishing procedures, which facilitate pigment adsorption and chemical interaction with the ceramic surface. Consistent with previous reports, glazed specimens exhibited greater resistance to chemical degradation and maintained more stable surface characteristics following mouthrinse exposure (Hashim and Mansoor 2021; Singh et al. 2017).

All polished specimens exhibited surface roughness values exceeding the 0.2 µm threshold that has been associated with increased bacterial plaque retention (Bollenl et al. 1997; Younes et al. 2024). Clinically, these findings suggest that preserving the glazed surface whenever possible may reduce plaque accumulation and contribute to maintaining the long‐term surface integrity of ceramic restorations. The magnitude of surface roughness changes also varied among the ceramic materials evaluated, indicating that the response to mouthrinse exposure depends not only on the polishing procedure but also on the intrinsic properties of the ceramic material (Kanat‐Ertürk 2020; Bozkaya Bilgin and Tekçe 2025).

The present study has several notable strengths. Unlike many previous investigations that evaluated a single ceramic material or a limited number of mouthrinses, this study simultaneously compared four commonly used CAD/CAM ceramic materials, two clinically relevant surface conditions (glazed and polished), and three widely prescribed therapeutic mouthrinses using standardized experimental conditions. Furthermore, both color stability and surface roughness were evaluated following the same standardized immersion protocol for all experimental groups, allowing consistent comparisons among ceramic materials and surface treatments. Collectively, these methodological features provide a more comprehensive evaluation of material–mouthrinse interactions than many previously published in vitro studies (Alpkilic et al. 2021; Soygun et al. 2017; Esfahanizadeh et al. 2024).

Nevertheless, several limitations should be acknowledged. Only one commercial brand was evaluated for each ceramic category, and a single polishing system and immersion protocol were used. Surface roughness was assessed using *R*_a_ as the sole quantitative roughness parameter. Although *R*_a_ is a widely used and standardized measure of average surface deviations, it does not fully describe the complexity of surface topography and may not capture other relevant features of surface irregularities. The use of additional roughness parameters, such as *R*_z_, as well as three‐dimensional surface topography analysis, could provide a more comprehensive characterization of surface changes following mouthrinse exposure. In addition, the experimental design did not reproduce important intraoral variables, including thermal cycling, masticatory loading, salivary proteins, biofilm formation, toothbrushing abrasion, or restoration cementation, all of which may influence the long‐term clinical behavior of ceramic restorations. Accordingly, the present findings should be interpreted within the limitations of this in vitro design, and future studies incorporating comprehensive aging protocols and well‐designed clinical trials are warranted to validate these results.

## 5 Conclusion

Within the limitations of this in vitro study, the effects of therapeutic mouthrinses on the color stability and surface roughness of CAD/CAM ceramics depended on both ceramic composition and surface finish. Polished specimens generally exhibited greater increases in surface roughness than glazed specimens, regardless of ceramic type. Essential oil‐based mouthrinse produced the greatest color changes in several ceramic materials, whereas the effects of chlorhexidine and stannous fluoride varied according to ceramic composition. These findings suggest that both ceramic composition and surface finishing should be considered when selecting CAD/CAM restorative materials and recommending long‐term therapeutic mouthrinse use for patients with ceramic restorations. Further laboratory investigations incorporating clinically relevant aging protocols, followed by well‐designed clinical studies, are warranted to confirm these findings.

## Author Contributions

Fateme Ghasemian Gorji conducted the experimental work, collected data, drafted and edited the manuscript. Fariba Ezoji developed the methodology, supervised the project, and revised the manuscript. Ghazaleh Ahmadizenouz contributed to the conceptualization and methodology of the study. Seyedali Seyedmajidia contributed to formal data analysis and manuscript editing. All authors reviewed and approved the final version.

## Ethics Statement

The study protocol was approved by the Ethics Committee of Babol University of Medical Sciences (IR.MUBABOL.REC.1402.131).

## Conflicts of Interest

The authors declare no conflicts of interest.

## Data Availability

The data are available upon reasonable request from the corresponding author.
